# The Determination Method of Satisfactory Consistency of the Interval Number Pairwise Comparisons Matrix Based on Submatrix

**DOI:** 10.3390/e24121795

**Published:** 2022-12-08

**Authors:** Fengxia Jin, Zhonghua Wu, Kun Zhao, Juan L. G. Guirao, Huatao Chen

**Affiliations:** 1 School of Mathematical Sciences, Henan Institute of Science and Technology, Xinxiang 453003, China; 2 Taiyuan Satellite Launch Center, Taiyuan 030027, China; 3 Beijing Electro-Mechanical Engineering Institute, Beijing 100074, China; 4 Department of Applied Mathematics and Statistics, Technical University of Cartagena, Hospital de Marina, 30203 Cartagena, Spain; 5 Department of Mathematics, Faculty of Science, King Abdulaziz University, P.O. Box 80203, Jeddah 21589, Saudi Arabia; 6 Division of Dynamics and Control, School of Mathematics and Statistics, Shandong University of Technology, Zibo 255000, China

**Keywords:** interval number pairwise comparisons matrix, satisfying consistency, permutation matrix, cyclic matrix

## Abstract

The decision-maker obtains the pairwise comparisons matrix by comparing two entities. In the process of comparing the two entities, the relationship between the two entities and other entities is not considered. In this way, the judgment may be illogical. This paper mainly studies the satisfactory consistency of the interval number pairwise comparisons matrix based on cyclic matrix. Firstly, the illogical judgment entity in the process of the decision-maker’s judgment is expressed by the cyclic matrix. There are three entities and four entities to form the cyclic matrix. The relationship and various forms of the cyclic cycle formed by the four entities and the three entities are discussed; then, the satisfactory consistency of the interval number pairwise comparisons matrix is determined by judging whether there is a cyclic matrix in the submatrix of the interval number pairwise comparisons matrix. Finally, two examples are given to verify the rationality and effectiveness of the method.

## 1. Introduction

In a practical multi-attribute decision-making problem, judgment will be affected subjectively and objectively. Sometimes it is not easy to use accurate judgment values to express the comparison results between schemes, and accurate values do not necessarily exist; therefore, in most cases, the judgment result given by decision-makers is often uncertain. Some scholars prefer to use an interval number or word representing the expression of their judgment result, such as a language phrase, probabilistic language term set, uncertain language term set, interval number, fuzzy number, interval rough number, etc. They can be used in algebraic properties, cyber–physical communication, etc. [1,2,3]. Among them, interval numbers can represent the results of the decision-maker’s comparison of the two entities. Compared with a single value, an interval number can better express the judgment of the decision-maker. If the decision-maker cannot give a single value under the condition of considering many factors, it is obviously more appropriate to use an interval number; for example, when consumers choose to buy a house, the location and price of the house are the two most important factors to consider. Interval numbers [2,4] are used to express the importance between location and house price, so it can be considered that the importance between location and price is not fixed in the process of purchase. When some positions are perfect, the price may be a little higher, but if the position is not satisfactory, the importance of the price must be shown, which can better show the actual situation.

Saaty and Vargas [4] proposed using the interval number pairwise comparisons matrix to represent the decision-maker’s judgment results of pairwise comparison of objects; then, some scholars presented the definition and determination method of the consistency of the interval number pairwise comparisons matrix. The complete consistency requirement of the interval number pairwise comparisons matrix is relatively high. The decision-maker should not only give the comparison relationship between two entities, but also meet the transitivity of the importance of entities in the overall comparison. The requirement of satisfactory consistency is that the advantages and disadvantages of entities can be judged according to the comparison results of two entities given by the decision-maker. There is no need to consider the transitivity of the degree of importance. The transitivity of the degree of importance means that the entity a is more important than the entity b and the entity b is more important than the entity c. The degree of importance of the entity a than the entity c is related to the degree of importance of the entity a than the entity b and the entity b than the entity c. Reference [5] analyzed and summarized the consistency definitions of the interval number pairwise comparisons matrix in the existing literature, points out the irrationality of some definitions, gives the definitions of complete consistency, strong consistency, and satisfactory consistency of interval number pairwise comparisons matrix, discusses the relationship between them, and gives the method of consistency discrimination. Reference [6] analyzes the main reasons affecting the inconsistency of pairwise comparisons matrix, and puts forward some new methods to improve the consistency of pairwise comparisons matrix. A new definition of acceptable consistency is proposed [7]. As part of research into the consistency determination method of interval number pairwise comparisons matrix, Arbel [8] proposed a linear programming model for calculating the priority of interval number pairwise comparisons matrix, and supplemented this method in reference [9]. Wang presented a two-stage logarithmic objective programming method [10]. Leung and Zhu used the concept of allowable deviation to calculate the ranking weight of interval number pairwise comparisons matrix [11,12,13,14,15,16,17]. Tan [18] proposed the consistency approximation method of interval number reciprocal pairwise comparisons matrix by solving the optimization model and consistency definition. Based on the geometric consistency index (GCI) and mathematical programming model, it can be determined whether the matrix has satisfactory consistency [19]. A conversion entity is proposed to effectively convert inconsistent comparison matrix into consistent comparison matrix. In order to consider consistency, uncertainty, and normality at the same time, a new definition of acceptable comparison matrix is proposed [20]. Based on the least square method, a model for determining the basic interval multiplicative weight of generalized interval multiplicative preference relationship is established, and its solution is obtained by using the Lagrange multiplier method. It is proved that any interval multiplication preference relationship with uncertainty does not have complete consistency algorithm on the interval [21,22,23,24,25,26,27]. Virgilio quantifies the preference relationship of interval multiplication based on the Hadamard dissimilarity operator according to the row geometric average method or the eigenvalue method. This method can maintain acceptable consistency and synthesize the derivation of reliable and consistent preference relationship of interval multiplication [28]. Based on the consistency property, Herrera Viedma proposed the additive transitivity of fuzzy preference relations, and proposed a method to construct consistent fuzzy preference relations from a set of preference data [29]. Koczkodaj puts forward the axiom for correcting the inconsistency index of the pairwise comparisons matrix, and proposes that the inconsistency of the sub matrix of the pairwise comparisons matrix will not be worse than that of the pairwise comparisons matrix [30]. Reference [31] judged the satisfactory consistency of the linguistic judgement matrix though the standard 0–1 arrangement matrix. Most of these methods need to establish planning models, and the calculation process is complex. When the pairwise comparisons matrix does not have satisfactory consistency, there is no way to find illogical objects [32,33,34,35,36,37].

Based on the research of interval number pairwise comparisons matrix in the existing literature, this paper gives the definition of satisfactory consistency of the interval number pairwise comparisons matrix and cyclic matrix formed with three or four entities. If the interval number pairwise comparisons matrix does not have satisfactory consistency, there must be illogical judgment, then the cyclic matrix can be expressed as an illogical judgment. This paper also proved that the cyclic matrix is the submatrix of the 0–1 central value preference relationship matrix. This method can achieve the result only by transformation of the matrix. It is not only simple in the judgment process, but also applicable to the interval number pairwise comparisons matrix with equivalent entity. If the interval number pairwise comparisons matrix has satisfactory consistency, the order of advantages and disadvantages of each entity can be given directly.

## 2. Basic Knowledge

**Definition** **1.** [38]* For digital judgment matrix $\bar{A}={\left({\bar{a}}_{ij}\right)}_{n\times n}$, if for all ∀i,j,k satisfy*

(1) $${\bar{a}}_{ij}={\bar{a}}_{ik}{\bar{a}}_{kj},$$

*the matrix $\bar{A}$ is said to be consistent*.

**Definition** **2.** [39] *Let $a_{ij}=[l_{ij},u_{ij}]$ be a bounded closed interval. If $l_{ij},u_{ij}\in R$, $a_{ij}=[l_{ij},u_{ij}]$ is called the interval number. All interval numbers on the set of real numbers R are recorded as $I_{R}$, that is, $I_{R}=\{[l_{ij},u_{ij}]|l_{ij}\leq u_{ij},l_{ij},u_{ij}\in R\}$.*

**Definition** **3.** [11] *There are two interval numbers,*

$$a_{1}=[l_{1},u_{1}],\;a_{2}=[l_{2},u_{2}],\;l_{1}≻0,\;l_{2}≻0$$

*then,*

$$\begin{array}{c} \;a_{1}\cdot a_{2}=[l_{1}\cdot l_{2},u_{1}\cdot u_{2}],\\ 1/a_{1}=[1/u_{1},1/l_{1}]. \end{array}$$

*In the existing literature, the consistency definition of interval number pairwise comparisons matrix has different forms.*

**Definition** **4.** [40] *$A={\left(a_{ij}\right)}_{n\times n}$ is called completely consistent interval number pairwise comparisons matrix. If for all ∀i≺j≺k, it has $a_{ij}=a_{ik}a_{kj}$.*

**Definition** **5.** [5] *$A={\left(a_{ij}\right)}_{n\times n}$ is called strong consistency interval number pairwise comparisons matrix. If the digital judgment matrix $\bar{A}={\left({\bar{a}}_{kl}\right)}_{n\times n}({\bar{a}}_{kl}\in [l_{kl},u_{kl}])$ is consistent for any real number ${\bar{a}}_{ij}\in [l_{ij},u_{ij}]$, where at that time $k=i,\;l=j,\;{\bar{a}}_{kl}={\bar{a}}_{ij}$.*

**Definition** **6.** [5] *The interval number pairwise comparisons matrix $A={\left(a_{ij}\right)}_{n\times n}$ is consistent. If there is a number judgment matrix $\bar{A}={\left({\bar{a}}_{ij}\right)}_{n\times n}({\bar{a}}_{ij}\in [l_{ij},u_{ij}])$ is consistency.*

It is also difficult for the interval number pairwise comparisons matrix given by the decision-maker to have complete consistency or strong consistency. The purpose of the decision-maker to give the pairwise comparisons matrix is to obtain their good and bad order on the premise. If the interval number pairwise comparisons matrix does not have complete consistency, but has other consistency, it can also obtain the good and bad order of each entity. The expected purpose of giving the pairwise comparisons matrix is achieved; therefore, this paper discusses the satisfactory consistency of the interval number pairwise comparisons matrix, and how to obtain the order of each entity. In fact, the consistency given above is also the satisfactory consistency mentioned in another reference. This paper also gives a definition of the satisfactory consistency of interval number pairwise comparisons matrix, and proves that the definition given in this paper is equivalent to Definition 6; in addition, it should be emphasized that according to the definition of the interval number pairwise comparisons matrix, the elements of the given interval number pairwise comparisons matrix meet the reciprocity, that is, the value of its elements is obtained based on the 1–9 scale method proposed by Saaty [11]. According to the practical significance of the 1–9 scale method, the values of interval numbers discussed in this paper are either below or above 1. This is because if the interval number [1/2, 4] is used to indicate the superiority of the entity i over the entity j, [1, 4] indicates that the entity i is superior to the entity j, and [1/2, 1] indicates that the entity j is worse than the entity i, resulting in contradictory results; therefore, the values of interval numbers are required to be either below or above 1.

**Definition** **7.** [11]* Let $a_{ij}=[l_{ij},u_{ij}]$ be an interval number, $m(a_{ij})=\frac{1}{2}(l_{ij}+u_{ij})$ is called the center of $a_{ij}=[l_{ij},u_{ij}]$.*

**Definition** **8.** *For the interval number pairwise comparisons matrix $A={\left(a_{ij}\right)}_{n\times n}$, if ∀i,k,j,∈I, where i≠k≠j,*

*(1)*  *when $m(a_{ik})≻1,\;m(a_{kj})\geq 1$ or $m(a_{ik})\geq 1,\;m(a_{kj})≻1$, $m(a_{ij})≻1$;*
*(2)*  *$m(a_{ik})=1,\;m(a_{kj})=1$ then $m(a_{ij})=1$;*

*it is said to $A={\left(a_{ij}\right)}_{n\times n}$ have satisfactory consistency.*

It is now proved that Definition 8 is equivalent to Definition 6.

**Proof.**  ⇒ if the interval number pairwise comparisons matrix has satisfactory consistency, then the digital judgment matrix $\bar{A}={\left({\bar{a}}_{ij}\right)}_{n\times n}({\bar{a}}_{ij}\in [l_{ij},u_{ij}])$ has consistency. According to the definition of the consistency of the digital judgment matrix, its elements meet ${\bar{a}}_{ij}={\bar{a}}_{ik}{\bar{a}}_{kj}$ $\left({\bar{a}}_{ik}\in [l_{ik},u_{ik}]\right)$. If $m(a_{ik})≻1$ then ${\bar{a}}_{ik}≻1$, it shows that the entity i is more important than the entity k. Similarly, if $m(a_{kj})\geq 1$, it can be deduced that the entity k is not worse than the entity j, that is, ${\bar{a}}_{kj}\geq 1$, $\bar{A}={\left({\bar{a}}_{ij}\right)}_{n\times n}({\bar{a}}_{ij}\in [l_{ij},u_{ij}])$ has consistency, and the ${\bar{a}}_{ij}={\bar{a}}_{ik}{\bar{a}}_{kj}$ deduced that the entity i is more important than the entity j, that is, $m({\bar{a}}_{ij})≻1$, it is proved to be true $m(a_{ik})\geq 1,\;m(a_{kj})≻1$ then $m(a_{ij})≻1$.

If $m(a_{ik})\geq 1,\;m(a_{kj})≻1$ then $m(a_{ij})≻1$ at that time $m(a_{ik})=1,\;m(a_{kj})=1$, then $m(a_{ij})=1$. It can be proved similarly, so it will not be repeated.

In this paper, the element of the interval number pairwise comparisons matrix meets either all below 1 or all above 1, so another proof method can be given.

⇒ In the interval number pairwise comparisons matrix $\bar{A}={\left({\bar{a}}_{ij}\right)}_{n\times n}({\bar{a}}_{ij}\in [l_{ij},u_{ij}])$, if the number pairwise comparisons matrix is consistent, the interval number pairwise comparisons matrix $A={\left(a_{ij}\right)}_{n\times n}$ is consistent, and if $\bar{A}={\left({\bar{a}}_{ij}\right)}_{n\times n}$ is consistent, it is satisfied ${\bar{a}}_{ij}={\bar{a}}_{ik}{\bar{a}}_{kj}{\bar{a}}_{ik}\in [l_{ik},u_{ik}]$. If $m(a_{ik})≻1$ then ${\bar{a}}_{ik}≻1$ ${\bar{a}}_{kj}\in [l_{kj},u_{kj}]$ (there are interval numbers below or above 1), if $m(a_{kj})\geq 1$, then ${\bar{a}}_{ij}={\bar{a}}_{ik}{\bar{a}}_{kj}$, ${\bar{a}}_{ij}≻1$, so $m(a_{ij})≻1$. It was established $m(a_{ij})≻1$ at that time $m(a_{ik})≻1$, $m(a_{kj})\geq 1$.

If $m(a_{ik})\geq 1,\;m(a_{kj})≻1$ then $m(a_{ij})≻1$ and when $m(a_{ik})=1,\;m(a_{kj})=1$ then $m(a_{ij})=1$. It can be proved similarly, so it will not be repeated.

⇐ For the interval number pairwise comparisons matrix $A={\left(a_{ij}\right)}_{n\times n}$, $a_{ij}=[l_{ij},u_{ij}]$, if ∀i,k,j,∈I, i≠k≠j where $m(a_{ik})≻1,$ $m(a_{kj})\geq 1$ satisfies $m(a_{ij})≻1$. When $m(a_{ik})≻1$, if you choose the appropriate ${\bar{a}}_{ik}\in [l_{ik},u_{ik}]$, you can deduce ${\bar{a}}_{ik}≻1$, and similar results ${\bar{a}}_{kj}\geq 1$ can be obtained. Let ${\bar{a}}_{ij}={\bar{a}}_{ik}{\bar{a}}_{kj}$, $m(a_{ij})≻1$ indicate that the entity i is superior to the entity j and there is a number in the existence of the entity $a_{ij}$ and is equal to ${\bar{a}}_{ij}$. The conditions of Definition 8 are ∀i,k,j,∈I and when $m(a_{ik})≻1,\;m(a_{kj})\geq 1$, $m(a_{ij})≻1$ for all i≠k≠j. Another explanation is that if these conditions are met, the sequence of advantages and disadvantages of entities can be obtained when comparing entities, and there will be no circulation; therefore, using this relationship, the comparison results of some entities are selected to construct a consistent digital pairwise comparisons matrix. In this way, when the interval number pairwise comparisons matrix meets the conditions of Definition 8, the digital pairwise comparisons matrix $\bar{A}={\left({\bar{a}}_{ij}\right)}_{n\times n}({\bar{a}}_{ij}\in [l_{ij},u_{ij}])$ is consistent, and it is concluded that the interval number pairwise comparisons matrix $A={\left(a_{ij}\right)}_{n\times n}$ is consistent, when $m(a_{ik})=1,\;m(a_{kj})=1$ and $m(a_{ij})=1$, it indicates that there is an equivalent entity.

When $m(a_{ik})\geq 1,\;m(a_{kj})≻1$ then $m(a_{ij})≻1$. It can be proved similarly, so it will not be repeated.  □

## 3. The Determination Method of Satisfactory Consistency of the Interval Number Pairwise Comparisons Matrix and the Ranking of Entities

### The Determination of Satisfactory Consistency of the Interval Number Pairwise Comparisons Matrix

**Definition** **9.** *$M={\left(m(a_{ij})\right)}_{n\times n}$ is called the central value matrix of the interval number pairwise comparisons matrix $A={\left(a_{ij}\right)}_{n\times n}$ ($a_{ij}=[l_{ij},u_{ij}]$), where:*

(2) $$m(a_{ij})=\frac{1}{2}(l_{ij}+u_{ij})$$

**Definition** **10.** *$W={\left(w_{ij}\right)}_{n\times n}$ is called the central value preference relation matrix of the interval number pairwise comparisons matrix $A={\left(a_{ij}\right)}_{n\times n}$ ($a_{ij}=[l_{ij},u_{ij}]$), where:*

(3) $$w_{ij}=\left\{\begin{array}{cc} 1 & m(a_{ij})\geq 1\\ 0 & m(a_{ij})<1 \end{array}\right.$$

**Definition** **11.** *In the center value preference relation matrix $Q={\left(q_{ij}\right)}_{n\times n}$, $a_{i}=\sum_{j=1}^{n}q_{ij}$ is the row preference value of row i of preference relation matrix; $b_{j}=\sum_{i=1}^{n}q_{ij}$ is the column preference value of column j of preference relation matrix.*

**Definition** **12.** *Let $W={\left(w_{ij}\right)}_{n\times n}$ be the center value preference relation matrix of the interval number matrix $A={\left(a_{ij}\right)}_{n\times n}$, and arrange the elements according to the number of preference values of the row. In order to ensure the original preference relation is also unchanged, the columns are adjusted accordingly. In this way, a new matrix $R={\left(r_{ij}\right)}_{n\times n}$ is obtained, which is called 0–1 center value permutation matrix of the interval number matrix.*

*For example:*

$$W=\begin{array}{cc} & \begin{array}{cccc} x_{1} & x_{2} & x_{3} & x_{4} \end{array}\\ \begin{array}{c} x_{1}\\ x_{2}\\ x_{3}\\ x_{4} \end{array} & \left(\begin{array}{cccc} 1 & \;0 & \;1 & 0\\ 1 & \;1 & \;1 & \;1\\ 0 & \;0 & \;1 & \;0\\ 1 & \;0 & \;1 & \;1 \end{array}\right) \end{array}\mathrm{then}\;R=\begin{array}{cc} & \begin{array}{cccc} x_{2} & x_{4} & x_{1} & x_{3} \end{array}\\ \begin{array}{c} x_{2}\\ x_{4}\\ x_{1}\\ x_{3} \end{array} & \left(\begin{array}{cccc} 1 & \;1 & \;1 & 1\\ 0 & \;1 & \;1 & \;1\\ 0 & \;0 & \;1 & \;1\\ 0 & \;0 & \;0 & \;1 \end{array}\right) \end{array}$$

**Definition** **13.** *In the submatrix of the 0–1 permutation preference relation matrix $R={\left(r_{ij}\right)}_{n\times n}$ of the interval number pairwise comparisons matrix $A={\left(a_{ij}\right)}_{n\times n}$, the form is as follows:*

$$\begin{array}{cccc} \begin{array}{cc} & \begin{array}{cc} x_{i} & x_{k} \end{array}\\ \begin{array}{c} x_{j}\\ x_{i} \end{array} & (\begin{array}{cc} 1 & \;0\\ 1 & \;1 \end{array}); \end{array} & \begin{array}{cc} & \begin{array}{cc} x_{k} & x_{i} \end{array}\\ \begin{array}{c} x_{i}\\ x_{j} \end{array} & (\begin{array}{cc} 1 & \;1\\ 0 & \;1 \end{array}); \end{array} & & \\ & & \begin{array}{cc} & \begin{array}{cc} x_{i} & x_{k} \end{array}\\ \begin{array}{c} x_{i}\\ x_{j} \end{array} & (\begin{array}{cc} 1 & \;1\\ 1 & \;0 \end{array}); \end{array} & \begin{array}{cc} & \begin{array}{cc} x_{k} & x_{i} \end{array}\\ \begin{array}{c} x_{j}\\ x_{i} \end{array} & (\begin{array}{cc} 0 & \;1\\ 1 & \;1 \end{array}), \end{array} \end{array}$$

*and the matrix in this form is called the cyclic matrix formed by the entities $x_{i},x_{j},x_{k}$.*

**Definition** **14.** *In the submatrix of the 0–1 permutation preference relation matrix $R={\left(r_{ij}\right)}_{n\times n}$ of the interval number pairwise comparisons matrix $A={\left(a_{ij}\right)}_{n\times n}$, the form is as follows:*

$$\begin{array}{ccc} \begin{array}{cc} & \begin{array}{ccc} x_{i} & x_{j} & x_{l} \end{array}\\ \begin{array}{c} x_{i}\\ x_{k} \end{array} & (\begin{array}{ccc} 1 & \;1 & \;0\\ 1 & \;0 & \;1 \end{array}); \end{array} & \begin{array}{cc} & \begin{array}{ccc} x_{i} & x_{l} & x_{j} \end{array}\\ \begin{array}{c} x_{i}\\ x_{k} \end{array} & (\begin{array}{ccc} 1 & \;0 & \;1\\ 1 & \;1 & \;0 \end{array}); \end{array} & \\ \begin{array}{cc} & \begin{array}{ccc} x_{j} & x_{i} & x_{l} \end{array}\\ \begin{array}{c} x_{i}\\ x_{k} \end{array} & (\begin{array}{ccc} 1 & \;1 & \;0\\ 0 & \;1 & \;1 \end{array}); \end{array} & \begin{array}{cc} & \begin{array}{ccc} x_{j} & x_{l} & x_{i} \end{array}\\ \begin{array}{c} x_{i}\\ x_{k} \end{array} & (\begin{array}{ccc} 1 & \;0 & \;1\\ 0 & \;1 & \;1 \end{array}); \end{array} & \\ & \begin{array}{cc} & \begin{array}{ccc} x_{l} & x_{i} & x_{j} \end{array}\\ \begin{array}{c} x_{i}\\ x_{k} \end{array} & (\begin{array}{ccc} 0 & \;1 & \;1\\ 1 & \;1 & \;0 \end{array}); \end{array} & \begin{array}{cc} & \begin{array}{ccc} x_{l} & x_{j} & x_{i} \end{array}\\ \begin{array}{c} x_{i}\\ x_{k} \end{array} & \left(\begin{array}{ccc} 0 & \;1 & \;1\\ 1 & \;0 & \;1 \end{array}\right) \end{array} \end{array}$$

*the matrix in this form is called the cyclic matrix formed by the entities $x_{i},x_{j},x_{k},x_{l}$.*

The cyclic matrix formed by three entities or four entities is a submatrix of the 0–1 permutation preference relationship matrix, and the reverse is not necessarily true. The cyclic matrix composed of four entities has more than the above six forms, because in the same interval number pairwise comparisons matrix, the row and column of each entity are fixed. In the above definition, they are only part of it, and there are the following forms,

$$\begin{array}{cc} \begin{array}{cc} & \begin{array}{ccc} x_{i} & x_{j} & x_{l} \end{array}\\ \begin{array}{c} x_{k}\\ x_{i} \end{array} & (\begin{array}{ccc} 1 & \;0 & \;1\\ 1 & \;1 & \;0 \end{array}); \end{array} & \begin{array}{cc} & \begin{array}{ccc} x_{i} & x_{l} & x_{j} \end{array}\\ \begin{array}{c} x_{k}\\ x_{i} \end{array} & (\begin{array}{ccc} 1 & \;1 & \;0\\ 1 & \;0 & \;1 \end{array}); \end{array}\\ \begin{array}{cc} & \begin{array}{ccc} x_{j} & x_{i} & x_{l} \end{array}\\ \begin{array}{c} x_{k}\\ x_{i} \end{array} & (\begin{array}{ccc} 0 & \;1 & \;1\\ 1 & \;1 & \;0 \end{array}); \end{array} & \begin{array}{cc} & \begin{array}{ccc} x_{j} & x_{l} & x_{i} \end{array}\\ \begin{array}{c} x_{k}\\ x_{i} \end{array} & (\begin{array}{ccc} 0 & \;1 & \;1\\ 1 & \;0 & \;1 \end{array}); \end{array}\\ \begin{array}{cc} & \begin{array}{ccc} x_{l} & x_{i} & x_{j} \end{array}\\ \begin{array}{c} x_{k}\\ x_{i} \end{array} & (\begin{array}{ccc} 1 & \;1 & \;0\\ 0 & \;1 & \;1 \end{array}); \end{array} & \begin{array}{cc} & \begin{array}{ccc} x_{l} & x_{j} & x_{i} \end{array}\\ \begin{array}{c} x_{k}\\ x_{i} \end{array} & (\begin{array}{ccc} 1 & \;0 & \;1\\ 0 & \;1 & \;1 \end{array}); \end{array} \end{array}$$

If the corresponding entities $x_{i},x_{j},x_{k}$ are selected as the row element, other forms of circular matrix can be formed. Although there are many forms, in essence, there are only six matrices. They are different in form and have the same substantive relationship, such as:

$$\begin{array}{cccccc} \begin{array}{cc} & \begin{array}{ccc} x_{i} & x_{j} & x_{l} \end{array}\\ \begin{array}{c} x_{i}\\ x_{k} \end{array} & \left(\begin{array}{ccc} 1 & \;1 & \;0\\ 1 & \;0 & \;1 \end{array}\right) \end{array} & \text{is the same as} & \begin{array}{cc} & \begin{array}{ccc} x_{i} & x_{l} & x_{j} \end{array}\\ \begin{array}{c} x_{k}\\ x_{i} \end{array} & (\begin{array}{ccc} 1 & \;1 & \;0\\ 1 & \;0 & \;1 \end{array}); \end{array} & \begin{array}{cc} & \begin{array}{ccc} x_{i} & x_{l} & x_{j} \end{array}\\ \begin{array}{c} x_{i}\\ x_{k} \end{array} & \left(\begin{array}{ccc} 1 & \;0 & \;1\\ 1 & \;1 & \;0 \end{array}\right) \end{array} & \text{is the same as} & \begin{array}{cc} & \begin{array}{ccc} x_{i} & x_{j} & x_{l} \end{array}\\ \begin{array}{c} x_{k}\\ x_{i} \end{array} & (\begin{array}{ccc} 1 & \;0 & \;1\\ 1 & \;1 & \;0 \end{array}); \end{array}\\ \begin{array}{cc} & \begin{array}{ccc} x_{j} & x_{i} & x_{l} \end{array}\\ \begin{array}{c} x_{i}\\ x_{k} \end{array} & \left(\begin{array}{ccc} 1 & \;1 & \;0\\ 0 & \;1 & \;1 \end{array}\right) \end{array} & \text{is the same as} & \begin{array}{cc} & \begin{array}{ccc} x_{l} & x_{i} & x_{j} \end{array}\\ \begin{array}{c} x_{k}\\ x_{i} \end{array} & (\begin{array}{ccc} 1 & \;1 & \;0\\ 0 & \;1 & \;1 \end{array}); \end{array} & \begin{array}{cc} & \begin{array}{ccc} x_{j} & x_{l} & x_{i} \end{array}\\ \begin{array}{c} x_{i}\\ x_{k} \end{array} & \left(\begin{array}{ccc} 1 & \;0 & \;1\\ 0 & \;1 & \;1 \end{array}\right) \end{array} & \text{is the same as} & \begin{array}{cc} & \begin{array}{ccc} x_{l} & x_{j} & x_{i} \end{array}\\ \begin{array}{c} x_{i}\\ x_{k} \end{array} & (\begin{array}{ccc} 1 & \;0 & \;1\\ 0 & \;1 & \;1 \end{array}); \end{array}\\ \begin{array}{cc} & \begin{array}{ccc} x_{l} & x_{i} & x_{j} \end{array}\\ \begin{array}{c} x_{i}\\ x_{k} \end{array} & \left(\begin{array}{ccc} 0 & \;1 & \;1\\ 1 & \;1 & \;0 \end{array}\right) \end{array} & \text{is the same as} & \begin{array}{cc} & \begin{array}{ccc} x_{j} & x_{i} & x_{l} \end{array}\\ \begin{array}{c} x_{k}\\ x_{i} \end{array} & (\begin{array}{ccc} 0 & \;1 & \;1\\ 1 & \;1 & \;0 \end{array}); \end{array} & \begin{array}{cc} & \begin{array}{ccc} x_{l} & x_{j} & x_{i} \end{array}\\ \begin{array}{c} x_{i}\\ x_{k} \end{array} & \left(\begin{array}{ccc} 0 & \;1 & \;1\\ 1 & \;0 & \;1 \end{array}\right) \end{array} & \text{is the same as} & \begin{array}{cc} & \begin{array}{ccc} x_{j} & x_{i} & x_{l} \end{array}\\ \begin{array}{c} x_{i}\\ x_{k} \end{array} & (\begin{array}{ccc} 0 & \;1 & \;1\\ 1 & \;0 & \;1 \end{array}); \end{array} \end{array}$$

There can be many forms, but there are actually six forms; therefore, if the selected submatrix formed by two rows and three columns is any of the above six forms, the corresponding entities for forming a cycle can be found.

The cycle formed by the four entities includes the cycle formed by the three entities. Because the comparison relationship between the four entities is not transitive, the comparison relationship between the three entities is not transitive. The cyclic matrix formed by the four entities includes the cyclic matrix formed by the three entities; however, the comparison relationship of any three of four entities will not form a circle.

**Theorem** **1.** *The necessary and sufficient condition for the interval number pairwise comparisons matrix $A={\left(a_{ij}\right)}_{n\times n}$ to have satisfactory consistency is that there is no cyclic matrix formed by three or four entities in the submatrix of its 0–1 arrangement preference relationship matrix $R={\left(r_{ij}\right)}_{n\times n}$.*

**Proof.**  Sufficiency. No matter how many entities form a circle, it will eventually include three or four entities. The judgment of the entity corresponding to the cycle is illogical, which affects the ranking of all entities. Due to illogical judgment, the interval number pairwise comparisons matrix does not have satisfactory consistency. If there is no form of the cycle matrix defined above in any sub matrix of the 0–1 permutation preference relationship matrix of the interval number pairwise comparisons matrix $A={\left(a_{ij}\right)}_{n\times n}$, it indicates that there is no cycle circle formed by three or four entities in the judgment result given by the decision-maker, and there is no cycle formed by more entities; then the comparison results of various entities are transitive as a whole. It can be seen that the interval number pairwise comparisons matrix $A={\left(a_{ij}\right)}_{n\times n}$ has satisfactory consistency.

Necessity. If the interval number pairwise comparisons matrix $A={\left(a_{ij}\right)}_{n\times n}$ has satisfactory consistency, the ranking of elements can be obtained according to the pairwise comparisons matrix given by the decision-maker, and the corresponding 0–1 arrangement preference relationship matrix is an upper triangular matrix. Because the advantages and disadvantages of the optimal element compared with other elements should be above 1, the corresponding elements in the first row of the arranged matrix should be 1. By analogy, the matrix after the final arrangement should be the upper triangular matrix; on the contrary, if the element in the lower triangular matrix of the corresponding 0–1 arrangement preference relationship matrix is not 0; that is, there is an element of 1, and this element affects the transitivity of the comparison relationship of the whole elements. If this element is $r_{ki}=1$, and $r_{ik}=0$, it means that at least a 1 is missing after the main diagonal element, and the matrix is arranged according to the number of 1. If there is no 1 in front of the main diagonal of the row, there will be fewer 1 below the row i. Let the element be $r_{ip},\cdots ,r_{iq}$ behind the main diagonal of the row i. Now, we know that the elements $r_{kp},\cdots ,r_{kq}$ corresponding to the row k are all 1. In this way, the number of 1 in the elements $r_{ip},\cdots ,r_{iq}$ is less than that in the elements $r_{kp},\cdots ,r_{kq}$; therefore, there must be 1 in front of $r_{ii}$ or there must be 0 in the elements $r_{kp},\cdots ,r_{kq}$. We will discuss this according to the situation.

If there is 1 in front of $r_{ii}$ and $r_{ih}=1$, if the element corresponding in the row k is $r_{kh}$, if $r_{kh}=0$, the cycle matrix $\begin{array}{cc} & \begin{array}{cc} r_{h} & r_{i} \end{array}\\ \begin{array}{c} x_{i}\\ x_{k} \end{array} & \left(\begin{array}{cc} 1 & \;1\\ 0 & \;1 \end{array}\right) \end{array}$ can be formed corresponding to three entities. If there is 0 in the elements of $r_{ip},\cdots ,r_{iq}$, if $r_{ij}=0$, then $r_{kj}=1$, the cyclic matrix $\begin{array}{cc} & \begin{array}{ccc} x_{h} & x_{i} & x_{j} \end{array}\\ \begin{array}{c} x_{i}\\ x_{k} \end{array} & \left(\begin{array}{ccc} 1 & \;1 & \;0\\ 0 & \;1 & \;1 \end{array}\right) \end{array}$ can be formed by four entities. Suppose that there is an element with 0 in front of $r_{ii}$, if $r_{ig}=0$ and $r_{kg}=1$, it can form the cyclic matrix $\begin{array}{cc} & \begin{array}{ccc} x_{g} & x_{h} & x_{i} \end{array}\\ \begin{array}{c} x_{i}\\ x_{k} \end{array} & \left(\begin{array}{ccc} 0 & \;1 & \;1\\ 1 & \;0 & \;1 \end{array}\right) \end{array}$ by four entities. Assuming that there is no element with 1 in the front of $r_{ii}$, no matter whether there is an element with 1 in the front of $r_{ki}$ or not, there must be an element with 0 in $r_{kp},\cdots ,r_{kq}$. If $r_{kj}=0$, there is a cycle matrix $\begin{array}{cc} & \begin{array}{cc} r_{i} & r_{j} \end{array}\\ \begin{array}{c} x_{i}\\ x_{k} \end{array} & \left(\begin{array}{cc} 1 & \;1\\ 1 & \;0 \end{array}\right) \end{array}$ formed by $r_{i},r_{j},r_{k}$. If $r_{il}=0$ behind the element $r_{ii}$ in the row i and $r_{kl}=1$, there is a cycle matrix $\begin{array}{cc} & \begin{array}{ccc} x_{i} & x_{j} & x_{l} \end{array}\\ \begin{array}{c} x_{i}\\ x_{k} \end{array} & \left(\begin{array}{ccc} 1 & \;1 & \;0\\ 1 & \;0 & \;1 \end{array}\right) \end{array}$ formed by four entities. If there is an element with 0 in front of the element $r_{ii}$, it is assumed that $r_{ig}=0$, and there is a cyclic matrix $\begin{array}{cc} & \begin{array}{ccc} x_{g} & x_{i} & x_{j} \end{array}\\ \begin{array}{c} x_{i}\\ x_{k} \end{array} & \left(\begin{array}{ccc} 0 & \;1 & \;1\\ 1 & \;1 & \;0 \end{array}\right) \end{array}$ formed by four entities.

Now, let us address the case of $r_{ik}=1$ and $r_{ii}=1$, which are all in line of i, and the cyclic matrix containing $r_{ik}=1$ and $r_{ii}=1$ are $\begin{array}{cc} & \begin{array}{cc} r_{i} & r_{k} \end{array}\\ \begin{array}{c} x_{i}\\ x_{j} \end{array} & \left(\begin{array}{cc} 1 & \;1\\ 1 & \;0 \end{array}\right) \end{array}$ and $\begin{array}{cc} & \begin{array}{cc} r_{i} & r_{k} \end{array}\\ \begin{array}{c} x_{j}\\ x_{i} \end{array} & \left(\begin{array}{cc} 1 & \;0\\ 1 & \;1 \end{array}\right) \end{array}$, which are formed by three entities. In the corresponding line of i, above $r_{ii}=1$, are $r_{mi},\cdots ,r_{ni}$ and the values are all 1. It is assumed that the above elements of $r_{ik}=1$ in the column k are $r_{mk},\cdots ,r_{nk}$. If the values of $r_{mk},\cdots ,r_{nk}$ are all 1, it is assumed that the elements below $r_{ii}$ in the column i appear $r_{ji}=1$. If $r_{jk}=0$, then $r_{ji}=1$, $r_{jk}=0$, $r_{ik}=1$, and $r_{ii}=1$, which form the cyclic matrix $\begin{array}{cc} & \begin{array}{ccc} x_{i} & x_{k} & x_{l} \end{array}\\ \begin{array}{c} x_{i}\\ x_{j} \end{array} & \left(\begin{array}{ccc} 1 & \;1 & \;0\\ 1 & \;0 & \;1 \end{array}\right) \end{array}$; If there are other elements in the row i and k that are 0, it is assumed that $r_{ig}=0$ and $r_{kg}=0$, and there is a circular circle matrix $\begin{array}{cc} & \begin{array}{ccc} x_{i} & x_{g} & x_{k} \end{array}\\ \begin{array}{c} x_{i}\\ x_{j} \end{array} & \left(\begin{array}{ccc} 1 & \;0 & \;1\\ 1 & \;1 & \;0 \end{array}\right) \end{array}$ formed by four entities. If $r_{ji}=1$ and $r_{jk}=1$, then the number of 1 in the column of k is more than in the column of i. This is unreasonable in the permutation matrix; then, there must be an element with 0 in the $r_{mk},\cdots ,r_{nk}$, set $r_{lk}=0$, and there is a cyclic matrix $\begin{array}{cc} & \begin{array}{cc} r_{k} & r_{i} \end{array}\\ \begin{array}{c} x_{l}\\ x_{i} \end{array} & \left(\begin{array}{cc} 0 & \;1\\ 1 & \;1 \end{array}\right) \end{array}$ corresponding to the three entities. If there is $r_{lp}=1$ and $r_{lp}=0$ after the row l and the column i, there is a cyclic matrix $\begin{array}{cc} & \begin{array}{ccc} x_{k} & x_{i} & x_{p} \end{array}\\ \begin{array}{c} x_{l}\\ x_{i} \end{array} & \left(\begin{array}{ccc} 0 & \;1 & \;1\\ 1 & \;1 & \;0 \end{array}\right) \end{array}$ formed by four entities. If there is $r_{lh}=1$ and $r_{ih}=0$ in front of the row l and column k, there is a cycle matrix $\begin{array}{cc} & \begin{array}{ccc} x_{h} & x_{k} & x_{l} \end{array}\\ \begin{array}{c} x_{l}\\ x_{i} \end{array} & \left(\begin{array}{ccc} 1 & \;0 & \;1\\ 0 & \;1 & \;1 \end{array}\right) \end{array}$ formed by four entities. If none of the elements below $r_{ii}=1$ in the column i is 1, and if all of them $r_{mk},\cdots ,r_{nk}$ are 1, then there are more 1 in the column k than in the column i, which does not conform to the fact that the number of 1 in the column k is less than or equal to the number of 1 in the column i, so there must be 0 elements in the $r_{mk},\cdots ,r_{nk}$. Let $r_{lk}=0$, then the cycle matrix $\begin{array}{cc} & \begin{array}{cc} r_{k} & r_{i} \end{array}\\ \begin{array}{c} x_{l}\\ x_{i} \end{array} & \left(\begin{array}{cc} 0 & \;1\\ 1 & \;1 \end{array}\right) \end{array}$ corresponding to the three entities of the form is formed. If it exists $r_{lp}=1$ and $r_{ip}=0$ below the row l and column k, then the cycle matrix $\begin{array}{cc} & \begin{array}{ccc} x_{k} & x_{i} & x_{p} \end{array}\\ \begin{array}{c} x_{l}\\ x_{i} \end{array} & \left(\begin{array}{ccc} 0 & \;1 & \;1\\ 1 & \;1 & \;0 \end{array}\right) \end{array}$ formed by the four entities of the form is formed. If there are $r_{lh}=1$ and $r_{ih}=0$ after the row l and column k, the cycle matrix $\begin{array}{cc} & \begin{array}{ccc} x_{h} & x_{k} & x_{i} \end{array}\\ \begin{array}{c} x_{l}\\ x_{i} \end{array} & \left(\begin{array}{ccc} 1 & \;0 & \;1\\ 0 & \;1 & \;1 \end{array}\right) \end{array}$ formed by four entities in the form is formed.  □

This theorem is proved. This theorem not only provides the method to judge whether the interval number pairwise comparisons matrix has satisfactory consistency, but also provides the method to find out the unreasonable entities. If the interval number pairwise comparisons matrix does not have satisfactory consistency, the cycle formed by three entities or four entities can be found.

Based on the above theorem, we can judge whether the interval number pairwise comparisons matrix has satisfactory consistency. The specific steps are as follows:

Step 1: provide the central value matrix corresponding to the pairwise comparisons matrix;

Step 2: provide the central value preference relationship matrix corresponding to the pairwise comparisons matrix;

Step 3: provide the 0–1 central value arrangement matrix corresponding to the judgment matrix;

Step 4: judge whether the 0–1 central value permutation matrix is a standard 0–1 permutation matrix;

Step 5: if the judgment in the previous step is yes, the judgment matrix has satisfactory consistency, the ranking of entities is given, and the judgment is finished;

Step 6: judge whether in the previous step the judgment matrix does not have satisfactory consistency, and, if so, proceed to the next step;

Step 7: judge the end.

## 4. Example Analysis

**Example** **1.** *Assumes that the interval number judgment matrix of four entities is provided. For convenience, these four entities are represented by $X=\{x_{1},x_{2},x_{3},x_{4}\}$,*

$$A=\begin{array}{cc} & \begin{array}{cccc} \;x_{1}\; & \;x_{2}\; & \;x_{3}\; & \;x_{4}\; \end{array}\\ \begin{array}{c} x_{1}\\ x_{2}\\ x_{3}\\ x_{4} \end{array} & \left(\begin{array}{cccc} 1 & \left[2,5\right] & \left[2,4\right] & \left[1,3\right]\\ \left[1/5,1/2\right] & 1 & \left[1,3\right] & \left[1,2\right]\\ \left[1/4,1/2\right] & \left[1/3,1\right] & 1 & \left[1/2,1\right]\\ \left[1/3,1\right] & \left[1/2,1\right] & \left[1,2\right] & 1 \end{array}\right) \end{array}$$

*Judge the satisfactory consistency of the interval number judgment matrix A. If it has satisfactory consistency, provide the order of advantages and disadvantages of the entities.*

Judge the judgment matrix according to the above judgment steps.

Step 1:

$$W=\begin{array}{cc} & \begin{array}{cccc} x_{1} & x_{2} & x_{3} & x_{4} \end{array}\\ \begin{array}{c} x_{1}\\ x_{2}\\ x_{3}\\ x_{4} \end{array} & \left(\begin{array}{cccc} \;1\; & \;1\; & \;1\; & \;1\;\\ 0 & 1 & 1 & 1\\ 0 & 0 & 1 & 0\\ 0 & 0 & 1 & 1 \end{array}\right) \end{array}$$

Step 2:

$$R=\begin{array}{cc} & \begin{array}{cccc} x_{1} & x_{2} & x_{4} & x_{3} \end{array}\\ \begin{array}{c} x_{1}\\ x_{2}\\ x_{4}\\ x_{3} \end{array} & \left(\begin{array}{cccc} \;1\; & \;1\; & \;1\; & \;1\;\\ 0 & 1 & 1 & 1\\ 0 & 0 & 1 & 1\\ 0 & 0 & 0 & 1 \end{array}\right) \end{array}$$

Step 3: according to the definition of standard 0–1 permutation matrix, it is concluded that R is a standard 0–1 permutation matrix, so A has satisfactory consistency, and the arrangement order of the entity is $x_{1}≻x_{2}≻x_{4}≻x_{3}$.

**Example** **2.** *There is a judgment matrix of four entities, which is used $X=\{x_{1},x_{2},x_{3},x_{4}\}$ to represent these four decision entities:*

$$A=\begin{array}{cc} & \begin{array}{cccc} \;x_{1}\; & \;x_{2}\; & \;x_{3}\; & \;x_{4}\; \end{array}\\ \begin{array}{c} x_{1}\\ x_{2}\\ x_{3}\\ x_{4} \end{array} & \left(\begin{array}{cccc} 1 & \left[2,4\right] & \left[3,5\right] & \left[1/5,1/3\right]\\ \left[1/4,1/2\right] & 1 & \left[1/2,1\right] & \left[2,5\right]\\ \left[1/5,1/3\right] & \left[1,2\right] & 1 & \left[1/3,1\right]\\ \left[3,5\right] & \left[1/5,1/2\right] & \left[1,3\right] & 1 \end{array}\right) \end{array}$$

*Judge the satisfactory consistency of the interval number pairwise comparisons matrix A. If it has satisfactory consistency, provide the order of advantages and disadvantages of the entity.*

Judge the pairwise comparisons matrix according to the above judgment steps.

Step 1:

$$W=\begin{array}{cc} & \begin{array}{cccc} \;x_{1}\; & \;x_{2}\; & \;x_{3}\; & \;x_{4}\; \end{array}\\ \begin{array}{c} x_{1}\\ x_{2}\\ x_{3}\\ x_{4} \end{array} & \left(\begin{array}{cccc} 1 & 3 & 4 & 4/15\\ 3/8 & 1 & 3/4 & 3.5\\ 4/15 & 1.5 & 1 & 2/3\\ 4 & 7/20 & 2 & 1 \end{array}\right) \end{array}$$

Step 2:

$$W=\begin{array}{cc} & \begin{array}{cccc} x_{1} & x_{2} & x_{3} & x_{4} \end{array}\\ \begin{array}{c} x_{1}\\ x_{2}\\ x_{3}\\ x_{4} \end{array} & \left(\begin{array}{cccc} \;1\; & \;1\; & \;1\; & \;0\;\\ 0 & 1 & 0 & 1\\ 0 & 1 & 1 & 0\\ 1 & 0 & 1 & 1 \end{array}\right) \end{array}$$

Step 3: the above matrix is not a standard 0–1 permutation matrix;

Step 4: $\begin{array}{cc} & \begin{array}{cc} x_{2} & x_{3} \end{array}\\ \begin{array}{c} x_{3}\\ x_{4} \end{array} & \left(\begin{array}{cc} 1 & \;1\\ 0 & \;1 \end{array}\right) \end{array}$ is a cycle matrix for the entities of $x_{2},x_{3},x_{4}$; $\begin{array}{cc} & \begin{array}{cc} x_{2} & x_{3} \end{array}\\ \begin{array}{c} x_{1}\\ x_{2} \end{array} & \left(\begin{array}{cc} 1 & \;1\\ 0 & \;1 \end{array}\right) \end{array}$ is a cycle matrix for the entities of $x_{1},x_{2},x_{3}$, $\begin{array}{cc} & \begin{array}{ccc} x_{1} & x_{2} & x_{3} \end{array}\\ \begin{array}{c} x_{3}\\ x_{4} \end{array} & \left(\begin{array}{ccc} 0 & \;1 & \;1\\ 1 & \;0 & \;1 \end{array}\right) \end{array}$ and $\begin{array}{cc} & \begin{array}{ccc} x_{2} & x_{3} & x_{4} \end{array}\\ \begin{array}{c} x_{1}\\ x_{2} \end{array} & \left(\begin{array}{ccc} 0 & \;1 & \;1\\ 1 & \;0 & \;1 \end{array}\right) \end{array}$ are cycle matrices for the entities of $x_{1},x_{2},x_{3},x_{4}$;

Step 5: the pairwise comparisons matrix does not have satisfactory consistency.

## 5. Conclusions

This paper mainly studies the satisfactory consistency determination method of interval number pairwise comparisons matrix based on submatrix. If the interval number pairwise comparisons matrix does not have satisfactory consistency, the illogical judgment formed can be found and represented by the submatrix of the interval number pairwise comparisons matrix. Only through observation and comparison can we find the entity of judging illogical. Further research is needed to correct the illogical pairwise comparisons.
